# The neural elements in the lining of the ventricular-subventricular zone: making an old story new by high-resolution scanning electron microscopy

**DOI:** 10.3389/fnana.2015.00134

**Published:** 2015-10-28

**Authors:** Carlos Alexandre dos Santos Haemmerle, Maria Inês Nogueira, Ii-sei Watanabe

**Affiliations:** ^1^Laboratory of Ultrastructure of Cells and Tissues, Department of Anatomy, Institute of Biomedical Sciences, University of São PauloSão Paulo, Brazil; ^2^Laboratory of Neurosciences, Department of Anatomy, Institute of Biomedical Sciences, University of São PauloSão Paulo, Brazil; ^3^Institute of Psychology, Center for Neuroscience and Behavior, University of São PauloSão Paulo, Brazil

**Keywords:** neurogenesis, lateral ventricle, axon, subventricular zone, olfactory bulb, ependymal cell, primary cilium

## Abstract

The classical description of the neural elements that compose the lining of brain ventricles introduces us to the single layer of ependymal cells. However, new findings, especially in the lateral ventricle (LV)—the major niche for the generation of new neurons in the adult brain—have provided information about additional cell elements that influence the organization of this part of the ventricular system and produce important contributions to neurogenesis. To complement the cell neurochemistry findings, we present a three-dimensional *in situ* description that demonstrates the anatomical details of the different types of ciliated cells and the innervation of these elements. After processing adult rat brains for ultrastructural analysis by high-resolution scanning electron microscopy (HRSEM) and transmission electron microscopy, we observed a heterogeneous pattern of cilia distribution at the different poles of the LV surface. Furthermore, we describe the particular three-dimensional aspects of the ciliated cells of the LV, in addition the fiber bundles and varicose axons surrounding these cells. Therefore, we provide a unique ultrastructural description of the three-dimensional *in situ* organization of the LV surface, highlighting its innervation, to corroborate the available neurochemical and functional findings regarding the factors that regulate this neurogenic niche.

## Introduction

The canonical description of the lining of brain ventricles emphasizes a particular class of supporting cells in the central nervous system that are arranged to form a single and continuous layer of multiciliated cells: the ependymal cells (Mitro and Palkovits, 1981; Bruni et al., 1985). However, new perspectives have recently been attributed on this layer, yielding new information about its organization and constituent elements, in relation to the different brain ventricles that comprise each part of the ventricular system (Mirzadeh et al., 2008; Del Bigio, 2010; Mullier et al., 2010).

The lateral ventricles (LVs) have been highlighted because they compose the major neurogenic niche of the adult brain: the ventricular-subventricular zone (V-SVZ; Altman, 1963; Takahashi et al., 1992; Chiasson et al., 1999; Tramontin et al., 2003; Coskun et al., 2008; Carlén et al., 2009; Chojnacki et al., 2009; Ihrie and Alvarez-Buylla, 2011). The neural elements at the lateral wall and rostral part of V-SVZ have been shown to influence the anatomical organization of this region into neurogenic spots. For instance, these areas contain: *(i)* biciliated ependymal cells (E2 cells) that have two motile cilia, as opposed to the classic multiciliated ependymal cells; and *(ii)* astrocytes (B1 cells), which act as primary neural progenitors and are characterized by a soma that can be found in the core of a pinwheel-like arrangement of ependymal cells and E2 cells. Furthermore, from this position, B1 cells can reach the LVs via a single cilium projecting from their apical surface (Doetsch et al., 1997; Mirzadeh et al., 2008, 2010a). In this way, at least three different cell types comprise the lining of the lateral wall of LVs.

The heterogeneity of the neural elements on the surface of the LVs also includes the type of innervation found on the ventricular surface, which consists of a dense plexus of varicose axons (Dinopoulos and Dori, 1995; Mikkelsen et al., 1997; Kim et al., 2010; Lennington et al., 2011). The ability to proliferate is intrinsic to the cells of the neurogenic niche, but the axonal signaling that occurs in the epithelium that lines the LV seems to play an important role in neurogenesis, as evidenced by its serotonergic innervation (Tong et al., 2014).

The identification of the neural elements that participate in the neurogenesis at the V-SVZ was achieved by genetic, neurochemical and transmission electron microscopy analysis. Moreover, high-resolution scanning electron microscopy (HRSEM) is a suitable and accurate anatomical approach for the investigation of all *in situ* aspects of each neural element and is not restricted to the neurochemistry of the element. To improve the detailed descriptions of the elements present on the lateral ventricular surface, the determination of the *in situ*, three-dimensional and ultrastructural aspects may enrich the current definition of the neural elements that participate in V-SVZ neurogenesis. Here, we present a description of the innervation upon the different ciliated elements that have previously been described on the lining of the surface of V-SVZ, to better demonstrate and to correlate them with the current functional data of the anatomical positions of regulatory elements of adult neurogenesis in the V-SVZ.

## Materials and Methods

### Animals

The animals were provided by the animal facility of the Department of Anatomy of the Institute of Biomedical Sciences, University of São Paulo, São Paulo (SP), Brazil. All procedures were performed in accordance with the Guidelines for the Care and Use of Mammals in Neuroscience and Behavior Research, which was established by the National Research Council according to the guidelines of the University of Sao Paulo, Institute of Biomedical Sciences Committee for Ethics and Animal Care in Experimental Research (protocol approval #128/2010).

We used Long-Evans rats (*Rattus norvegicus*) (*n* = 14, male, 320–360 g, 4–5 months old) housed in groups of five animals per plastic cage, with *ad libitum* access to water and commercial rat chow. The room temperature was controled at 21 ± 1°C, and the animals were kept on a 12/12 h light/dark cycle (lights on at 7:00 AM).

### Procedures for HRSEM

The animals (*n* = 10) were anesthetized by a single dose of 35% chloral hydrate solution (1 mL/animal). After the induction of anesthesia, the animals were perfused with 100 mL of 0.9% saline solution, which was followed by 400 mL of 2% paraformaldehyde plus 2.5% glutaraldehyde (Watanabe and Yamada, 1983) in 0.01 M sodium phosphate buffer (PBS) at pH 7.4. Each brain was removed; the LVs were dissected (Mirzadeh et al., 2010a) and immersed in the same fixative solution at room temperature for 3 h. Sequentially, the samples were washed in 0.01 M PBS (pH 7.4) and post-fixed in 2% OsO_4_, buffered in 0.01 M PBS (pH 7.4) for 2 h at 4°C. Then, the samples were washed and immersed in 0.01 M PBS (pH 7.4) overnight at 4°C.

Then, we washed the samples in de-ionized water and dehydrated them in ethanol solutions [70%, 80%, 90%, 95% (20 min) and 4 × 100% (1 h each)]. Sequentially, we dried the samples in a critical point device (Balzers CPD-030, Balzers—Liechtenstein) and mounted them on a metal base for further recovery with a layer of gold ions of 2 ηm thickness in an “Ion Sputter” device (Balzers—SCD-040, Balzers—Liechtenstein) (Tanaka, 1981; Tánaka, 1989; Watanabe et al., 1992).

### Procedures for TEM

The animals (*n* = 4) were anesthetized and perfused using the same method described in the previous section. The brains were removed and blocked to obtain only tissue from the periventricular area. Then, the samples were immersed in the same fixative solution (2% paraformaldehyde +2.5% glutaraldehyde in 0.1 M sodium cacodylate, pH 7.4) for 3 h at room temperature. Next, the samples were washed in 0.1 M PBS (pH 7.4) and cut into 100 μm sections in frontal plane on a vibratome. Post-fixation was performed in 1% OsO_4_ in 0.1 M PBS (pH 7.4) for 2 h at 4°C. After washing, the slices were immersed in 0.9% saline and 0.5% uranyl acetate solution for 8 h at room temperature. Then, the samples were dehydrated in ethanol solutions [70%, 80%, 90%, 95% (3 min) and 4 × 100% (3 min each)] and propylene oxide (2 × 3 min each). For the resin embedding process, the sections were first exposed to a mixture of 1:1 propylene oxide and Spurr resin (Electron Microscopy Sciences, USA) for 4 h at room temperature. Then, the samples were embedded in pure Spurr resin for 16 h. Sequentially, new Spurr resin was added, and the samples were covered by an acetate sheet for 72 h at 60°C, for complete resin polymerization.

The samples of the periventricular regions were glued to resin blocks and trimmed into areas of interest to produce semi-thin sections (400 ηm), using a glass knife in an ultra-microtome (Leica Ultracut UCT, Leica Microscopes AG, Vienna, Austria). The sections were mounted on histological slides, stained with 1% toluidine blue for observation by light microscopy, and cut into ultra-thin sections (60 ηm) with a diamond knife. We collected the sections in 150 mesh copper grids and counterstained them with saturated uranyl acetate and lead citrate (Watson, 1958; Reynolds, 1963; Watanabe and Yamada, 1983).

### Acquisition, Processing, Image Analysis and Quantification Process

The electron micrographs were obtained with a transmission electron microscope JEOL 1010, at 80 kV, located in the Institute of Biomedical Sciences of the University of São Paulo, and with a field emission scanning electron microscope JEOL JSM7401F, located at the Institute of Chemistry of the University of São Paulo. The settings of the acquired images were only adjusted to control the brightness, contrast and exposition levels. Furthermore, the coloring of the scanning electron micrographs was performed by the same researcher by zooming in and out to accurately identify the cell limits using Adobe Photoshop CS5 version 12.04 × 32 software (Adobe, San Jose, CA, USA).

To obtain the quantification data, we measured the length of each entirely visible cilium, the density of ciliary tufts that were clearly identified as multiciliated or biciliated tufts of ependymal cells and the single cilium of B1 cells. Three samples were analyzed from different areas of the LV surface, with respect to the rostrocaudal and dorsoventral levels. The data are represented as averages plus the standard deviation.

The anatomical references for the TEM samples obtained from the frontal sections were found in the rat brain atlas of Paxinos and Watson (1998).

## Results

**Figure 1:**
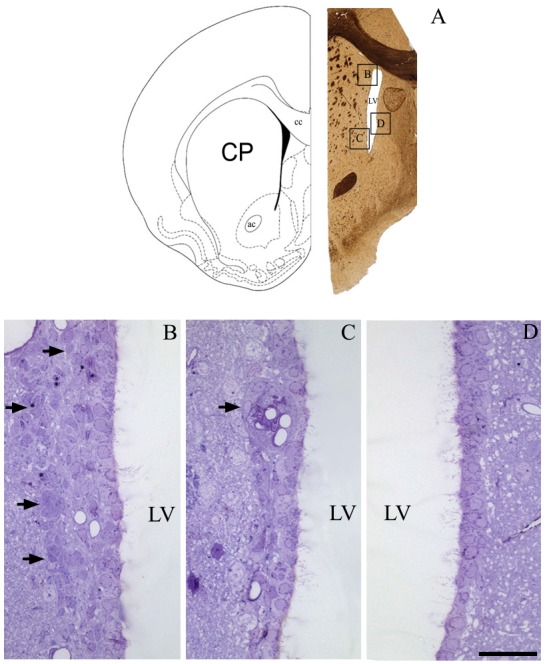
**The periventricular niche of the rat lateral ventricle (LV).** In **(A)**, a schematic drawing, adapted from Paxinos and Watson (1998), corresponding to 1.0 mm anterior to bregma that represents the frontal section obtained for a confirmation of cell types that comprises de adult ventricular-subventricular zone (V-SVZ), specifically at dorsal and ventral parts of lateral wall, and the medial wall. In **(B)**, arrows point to the migratory corridor in the dorsolateral SVZ. In **(C)**, arrow points to a characteristic islet, containing cells of SVZ parenchyma apposed to capillaries. In **(D)**, the typical pattern of lacking proliferative cells in the parenchyma of the medial wall of LV. Legend—ac, anterior commissure; cc, corpus callosum; CP, caudate-putamen; LV, lateral ventricle. Scale bar—**(B—D)** 100 μm.

To determine if our methods could contribute new information regarding the periventricular cellular niche, we first examined the rostral V-SVZ to confirm the efficiency of our protocol. Figure 1A shows an example image obtained at 1 mm anterior to the craniometric point bregma. As expected, the lining of LVs mainly consisted of a single layer of ciliated cells. The heterogeneity in the cellular composition of the V-SVZ was exclusively found at the lateral wall of the LV, and included the classic chain of migratory cells located adjacent to the blood vessels (Figures 1B,C). The parenchyma of the medial wall of the LV lacked the typical pattern of cells described for the lateral V-SVZ (Figure 1D).

**Figure 2:**
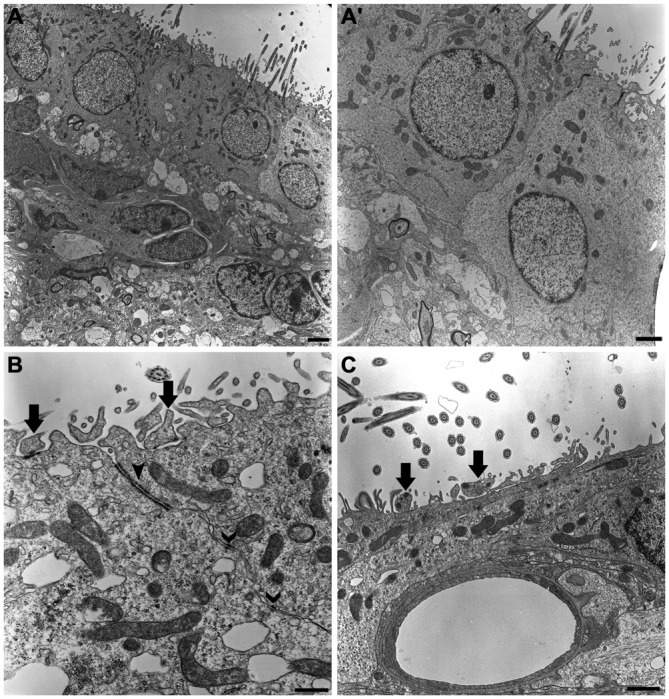
**The ultrastructure of the neurogenic niche of lateral ventricle.** Transmission electron photomicrographs showing the canonical and peculiar morphology of each cell that compose this niche. In subventricular zone, we corroborate the previous descriptions about the chain of proliferative and migratory neuronal precursors, with irregular nucleus in heterochromatin pattern and piriform or irregular shaped-cytoplasm **(A)**; we also confirm that the lining epithelia is predominantly characterized by the cuboidal ependymal cells, with their rounded or ovoid nucleus in euchromatin pattern, and a great density of mitochondria and cilia implantation in apical surface **(A’)**. In **(B)**, tight junctions (closed arrowheads) and desmosomes (open arrowheads) make the cell-cell adhesions to constitute the single cell layer of the lining epithelia. Supra-ependymal axons, characterized by mitochondria and synaptic vesicles are found running on apical surface of cells (arrows in **B,C**), also establishing synapsis with the adjoin cell, as revealed by the electron-dense dots pointed by arrows on axons in **(B)**. In **(C)**, note there is no electron-dense element between the axon and apical surface of the cell, suggesting the lack of a synaptic contact. Scale bars—**(A)**: 2 μm; **(A’)**: 1 μm; **(B)**: 600 ηm; **(C)**: 500 ηm.

Our ultrastructural description confirmed that multiciliated ependymal cells predominantly comprise the lining of the lateral wall of the LVs. Below the ependymal layer, we also observed cells in the subventricular zone that formed a chain of proliferative and migratory neuronal precursors (Figure 2A). The ependymal cells presented the following peculiar ultrastructural characteristics: *(i)* rounded or ovoid nuclei in a euchromatin pattern; *(ii)* a cuboidal or rounded plasmalemma; *(iii)* canonical mobile cilium with the 9:2 pattern of microtubule arrangement and respective basal bodies; and *(iv)* a high concentration of mitochondria at the apical surface of the cell (Figure 2A’). The adhesion of a single layer of ependymal cells was achieved via tight junctions and desmosomes located on the apical and lateral surfaces, respectively (Figure 2B). Cells that exhibited an irregular plasmalemma and radial cytoplasmic projections were occasionally observed in the lining of the LV and were in contact with capillaries (Figure 2C).

**Figure 3:**
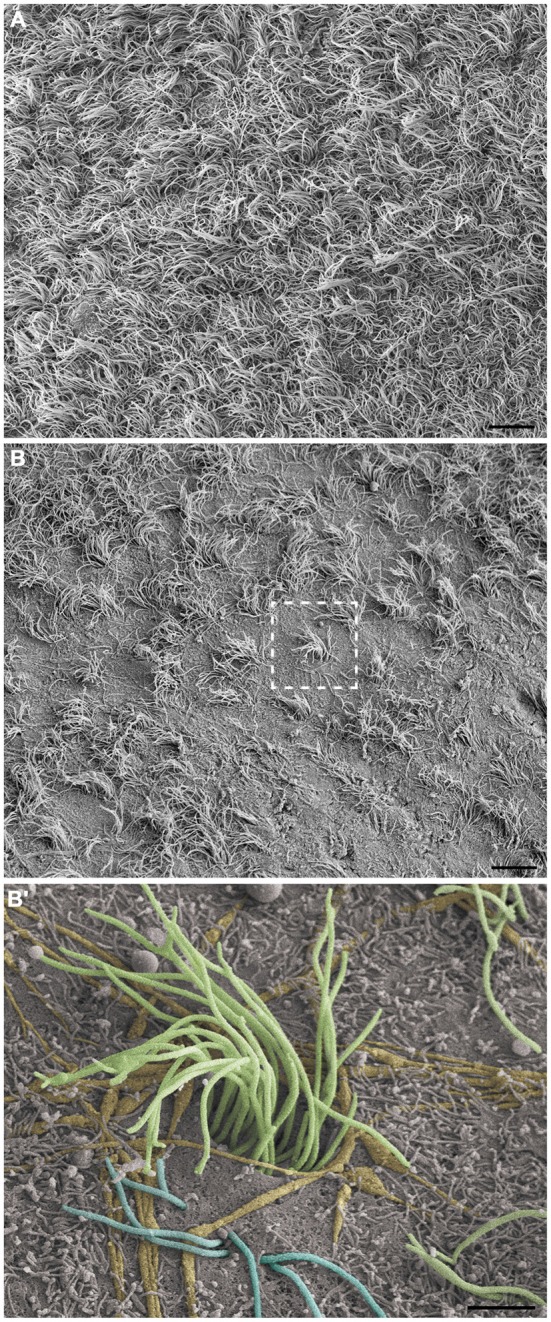
**Observation of the LV lining by high-resolution scanning electron microscopy (HRSEM).** Photomicrographs from the ependymal surface of the lateral wall of the LV of the rat brain, where it is possible to observe the territories with either high-density cilia, especially with a high length **(A)** or with low-density cilia, especially with a short length and including bare zones **(B)**. In **(B’)**, a magnification of the boxed area in **(B)**, the ependymal layer elements can be observed by its tridimensional and *in situ* details: the tuft of a multiciliated ependymal cell (green), bi-ciliated ependymal cells (E2) and an unusual cell with three cilia emerging in its apical surface (blue), axonal bundles and fibers innervating them (yellow) and being embraced by microvilli (not colored). Scale bars—**(A,B)**: 10 μm; **(B’)**: 2 μm.

Cilia and microvilli were heterogeneously distributed throughout the ventricular surface, forming areas with high and low-density ciliary tufts (Figures 3A,B). Because the HRSEM approach does not permit visualization of the cellular borders to allow determination of the number of cells, we estimated the cellular density by visualization of ciliary tufts or a single cilium. The anterior region of V-SVZ contained high-density cells (14.5 multiciliated tufts/mm^2^) with a longer average cilia length (10.02 μm ± 1.54), while the posterior region showed low-density cells (10.5 multiciliated tufts/ mm^2^) with a shorter cilia length (6.5 μm ± 1.5). We found that the average length of motile cilia of the multiciliated ependymal cells was 9.29 μm ± 1.36 μm. When we examined the low-density ciliary areas, it was possible to visualize the three-dimensional *in situ* morphology of all elements comprising the ventricular lining. For instance, in Figure 3B’ we show in detail the entire emerging multiciliated tuft that characterizes ependymal cells; we also confirmed the occurrence of biciliated ependymal cells [(E2 cells) 5.38 cells/mm^2^] that show a particular arrangement of the two emerging cilia (6.285 μm ± 1.29) similar to the classic ependymal cell arrangement. These E2 cells represented 37% of the total identified multiciliated ependymal cells. The microvilli were the smallest projections of the epithelia; some of these projections surrounded the axons that innervate the ventricular epithelia.

**Figure 4:**
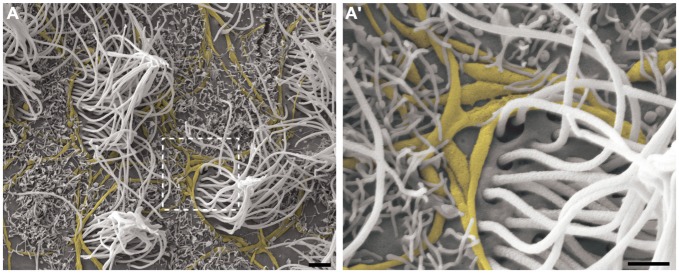
**The branching of axonal fibers on the ventricle lining.** High-resolution scanning electron photomicrographs of the lateral wall of LV, describing the varicosal branched axons (yellow) and its pattern of innervation surrounding the multi-ciliated ependymal cells (not-colored) **(A)**. Detailed description in **(A’)** shows the axonal varicosities close to the local of cilium emergence at apical surface of the cell. A large amount of microvilli are found recovering the lining, and embrace the axons in a way to putatively help attach them on the ventricular surface. Scale bars—**(A)**: 2 μm; **(A’)**: 1 μm.

As expected, long and varicose axonal projections were present on the ventricular surface. In the ultra-thin sections analyzed by TEM, we confirmed the presence of mitochondria and synaptic vesicles inside the axolemma (Figure 2C) and found evidence of electron-dense elements that characterize synapses between adjacent cells (Figure 2B). Using HRSEM, we clearly observed the three-dimensional aspects of fiber bundles branching in varicose axons that projected along the ventricular surface, particularly in those that innervated the tufts of cilia, and surrounding them at the ventricular floor (Figure 4A). Because varicosities have been found to contain synaptic vesicles, these axons may establish putative *en passant* synapses with the apical surface of the cells lining the epithelia (Figure 4A’).

**Figure 5:**
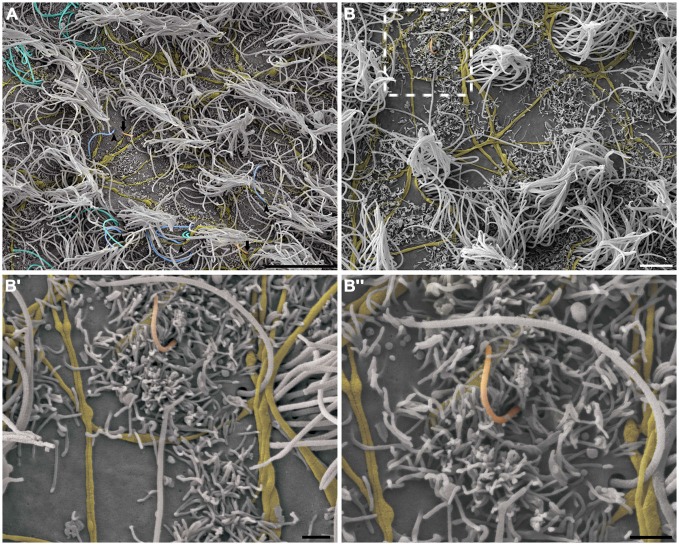
**The pattern of axonal branching and the innervation of single ciliated cells.** High-resolution scanning electron photomicrographs of the lateral wall of LV showing that the pattern of axonal branching (yellow) links different tufts of cilia (not-colored) and also aim the innervation of the short and single/primary cilium emerging at ventricular lumen—that characterizes the primary neural progenitor (peach-colored; arrows in **A** and detail in **B**). Progressive higher magnifications in **(B’)** and **(B”)** confirms the varicosal axonal fibers and the presence of a discrete collateral branch lying beside the short primary cilium emergence from apical surface. The unusual long length single cilia (blue-colored, arrow-heads) and bi-ciliated cells (cyan-colored) are showed in **(A)**. (Scale bars—**(A)**: 10 μm; **(B)**: 4 μm; **B’** e **B”**: 1 μm).

**Figure 6:**
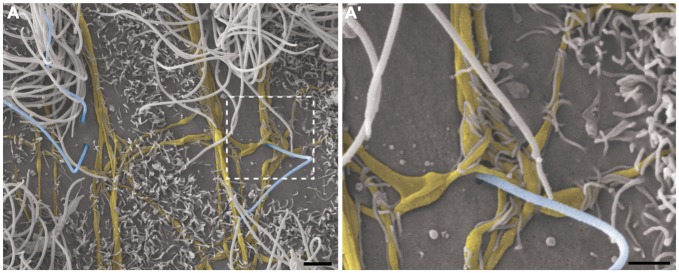
**Thick bundles of axonal fibers and the innervation of unusual long single ciliated cells.** High-resolution scanning electron photomicrographs of the lateral wall of lateral showing the inter-communication of two thick bundles of multiple axons (yellow) by collaterals and also the crossing of fibers in order to spread along the ventricular surface and innervate a long single-ciliated cell (blue; **A)**. In detail, the pattern of innervation surrounding the emergence local of the cilium; microvilli are also present in the apical surface of cells, wrapping the axonal fibers to facilitate their attachment on the ventricular floor **(A’)**. Scale bars—**(A)**: 2 μm; **(A’)**: 1 μm.

This pattern of axonal branching was also observed on the apical surface of cells that presented a short single/primary cilium (2.47 μm ± 0.72) (Figure 5). The presence of this feature is suggestive, but not conclusive, that these cells are astrocytes/primary neural progenitors that are located among the multiciliated ependymal cells, with a density of 1.61 short single cilium/mm^2^. Thick bundles containing multiple axonal fibers also exhibited collaterals (Figure 6A) that innervated long single cilia (7.6 μm ± 1.8), surrounding them at the point of emergence from the ventricular floor (Figure 6A’). Single-ciliated cells, regardless of the length of the cilium, comprised 29.6% of the identified multiciliated ependymal cells. However, when the cells were classified according to the length of the single cilium, the short single ciliated cells represented 11.1% of the multiciliated cells, while the long single ciliated cells constituted 18.5% of the identified multiciliated cells.

## Discussion

### Anatomical Aspects

Different characteristics have been proposed to differentiate each component of the brain ventricular system (Bruni et al., 1985; Mirzadeh et al., 2008; Mullier et al., 2010). In terms of the composition of the LV lining, our data corroborate that different ciliated elements and a specific cellular organization with respect to neurogenesis characterize the lateral wall of the LVs (Doetsch et al., 1997). Supported by a unique high-resolution, ultrastructural and three-dimensional descriptive methodology, this investigation supplements previous findings based on genetic approaches and staining of microtubule-constituting proteins that form the body of cilia and their implantation sites (as basal bodies) at the apical surface of different ciliated cells with specific morphologies, located in the lining of the V-SVZ (Mirzadeh et al., 2008).

The primary cilium of B1 cells has been characterized by its short length and internal organization of microtubules in a 9:0 pattern; it lacks motility compared to ependymal cells (Ibañez-Tallon et al., 2003; Tramontin et al., 2003). In regions with low-density of multiciliary tufts—where the long length cilia do not cover the small projections that also comprise the ventricle lining—we confirmed the existence of short primary cilia, including those with expected lengths, but intriguingly, we observed single cilia with long lengths. An exact neurochemical determination of cell borders and basal bodies, as described by Mirzadeh et al. (2010b), would help to determine if the long cilia are actually isolated cilia from a tuft that is located in a different pole of the cell. However, our intention was to achieve optimal preservation of brain structure for a high-resolution ultrastructural analysis that could provide support for future studies of putative transient phases related to the lengthening emergence or dislocation of primary cilia. In fact, it has been suggested that the average length of primary cilia can change, according to the different phases of the cell cycle (Avasthi and Marshall, 2012).

In contrast, the mobile cilia of ependymal cells have a 9:2 pattern of microtubule organization, which is designed for the coordinated beating that directs the flow of the cerebrospinal fluid (CSF; Breunig et al., 2010). Given the morphological complexity related to the sinuses and concave surfaces along the path of CSF flow, the multiciliated cells organize their polarity to correctly perform the beating that drives the CSF flow over the cavities of interventricular communication—the foramen of Monro—located close to the anterior part of V-SVZ (Guirao et al., 2010; Mirzadeh et al., 2010a; Kishimoto and Sawamoto, 2012). Investigations of the density of cilia on the ventricular surface should be supported by *in situ* methods, which will provide additional data to that obtained from reconstruction planes made from histological sectioning. In this manner, our data obtained by HRSEM provides a clear description of the distribution of cilia on the ventricular surface in high and low-density ciliated areas.

Regarding the different types of ciliated cells located on the surface of the LV, we verified that E2 cells (the biciliated ependymal cells) comprise this niche. These cells have been described as having two basal bodies and occasionally constituting the rosette-like or pinwheel organization in the mouse V-SVZ, representing a population of less than 5% of the cells that occupy the ventricular surface and surround the core formed by the B1-cells (the primary neural progenitors) (Mirzadeh et al., 2008). In terms of quantification, our data show a more substantial participation of these cells in the composition of V-SVZ (37%), at least in the low-density areas covered by long and multiple cilia from ependymal cells in adult rats.

Furthermore, our data are consistent with the presence of a dense innervation of the ventricular surface lining, characterized by bundles consisting of long axonal fibers that are highly branched and have varicosities along their axis, as reported by Richards et al. (1981) and Mikkelsen et al. (1997). The microvilli, further to cover the ventricular floor, are positioned to attach the axons to the ventricular epithelia, as previously shown by TEM (Tong et al., 2014). Our findings supplement this observation and provide a general description of the extension of the microvilli that wrap the axons that innervate the cells of the ventricular surface.

### Functional Aspects

Neural stem cells, also called primary neural progenitors, belong to the same cell lineage as the cells that initiate neurogenesis during embryonic development. A report based on fine ultrastructural analyses found that the primary neural progenitors in the adult brain are B1- astrocytes (Doetsch et al., 1999), which remain active in the adult V-SVZ; similar to B1 cells, radial astrocytes remain actives in the subgranular zone of dentate gyrus (Morshead et al., 1994; Doetsch et al., 1999; Kriegstein and Alvarez-Buylla, 2009; Fuentealba et al., 2012, 2015). In particular, B1 cells have a primary cilium that reaches the ventricle content, which is thought as a cellular “antenna” related to intracellular signaling events (Singla and Reiter, 2006; Goetz and Anderson, 2010; Molla-Herman et al., 2010; Farnum and Wilsman, 2011) that correspond to the paracrine signaling involved in the proliferation or initiation of cell turnover that generates neuroblasts (Plotnikova et al., 2009; Ihrie and Alvarez-Buylla, 2011). These neuroblasts enter the rostral migratory stream and travel toward the olfactory bulb, where they will become the new interneurons and integrate into the local circuitry (Alvarez-Buylla and Garcia-Verdugo, 2002; Merkle et al., 2014).

Mirzadeh et al. (2008) reported that B1 cells, which have a short single cilium, compose approximately one-third of the total population of cells that contact the CSF. We found a similar percentage of single ciliated cells, but we included cells with both short and long cilia. Although we examined the V-SVZ using a different animal model with a different age, it is important to note that primary cilia are present in quiescent cells and are resorbed by the cell before it enters the S-phase of cell proliferation. This event is dependent on Tctex-1, a light chain subunit of the dynein complex protein, in embryonic and adult brain development (Li et al., 2011; Avasthi and Marshall, 2012). Therefore, it may be possible that the different lengths of cilia observed may represent single ciliated cells in different phases of the mitotic cycle.

The biciliated cells located in the central canal play an important role in cell division and proliferation and the post-natal development of the spinal cord (Alfaro-Cervello et al., 2012). These biciliated cells located in the central canal have characteristics similar to the E2 cells in the LV, such as the 9:2 organization of motile cilia. However, it is important to note that E2 cells have not been described as dividing cells in the V-SVZ (Mirzadeh et al., 2008), and their function in neurogenesis is still unclear.

The axons present on the LV surface have been described by their immunoreactivity to serotonin, which specially influences neurogenesis by direct innervation of B1 astrocytes (Tong et al., 2014). However, other neuroactive substances, such as dopamine (Kim et al., 2010) and melanin-concentrated hormone (MCH; Haemmerle et al., 2015) have also been found in axons that innervate the regional domains of the V-SVZ. Regarding dopamine, it has been suggested that fibers originating from the substantia nigra and ventral tegmental area target respective domains in the V-SVZ, and its depletion impairs proliferation and neurogenesis in the adult brain (Höglinger et al., 2004, 2014; Kim et al., 2010; Lennington et al., 2011). MCH has been suggested to function as a regulator of the ciliary beating pace for the epithelium of the third ventricle (Conductier et al., 2013). However, due to the pattern of MCH innervation in the V-SVZ, future studies focusing on a putative role in neurogenesis must be conducted. Furthermore, catecholamines, GABA and glutamate circulate in the CSF in both normal and pathological conditions (Eckstein et al., 2008; Kovac et al., 2014). In this manner, the dense axonal plexus may represent a possible structural basis for the diversity of neuroactive substances released in the CSF that modulate neurogenesis in the V-SVZ (Bovetti et al., 2011; Pallotto and Deprez, 2014).

Therefore, using a unique methodology to describe the *in situ* and three-dimensional features of cell elements, we confirm that the LV lining does not merely consist of multiciliated ependymal cells, as claimed in the canonical description. It is important to highlight that this structure is comprised of biciliated cells and primary neural progenitors, each with a distinct ciliary apparatus. Furthermore, we corroborate previous observations regarding the innervation of the LV lining using a high-resolution methodology that visualized all elements present on the ventricular surface. The putative synaptic contacts involving axons are located in a position to exert functions previously proposed, such as the regulation of ciliary beating and neurogenesis in the V-SVZ. We believe that these data will provide an anatomical basis for the functional roles that have been proposed for the elements found in the lining of the LVs.

## Author Contributions

CH helped the design of the experiments, performed the tissue preparation, proceedings for high-resolution and transmission electron microscope methodology, processing of images, interpretation of data and helped to write the manuscript; MN was responsible for designing the experiments, interpretation of data and helped to write the manuscript; IW conceived the experiments, was responsible for images acquisition, data interpretation and manuscript preparation.

## Conflict of Interest Statement

The authors declare that the research was conducted in the absence of any commercial or financial relationships that could be construed as a potential conflict of interest.
